# Experiments and Observations on the Matter of Cancer, and on the Aërial Fluids Extricated from Animal Substances by Distillation and Putrefaction; Together with Some Remarks on Sulphureous Hepatic Air

**Published:** 1792

**Authors:** Adair Crawford


XIII. *Experiments and Obfervations on the Matter of Cancer, and on the aërial Fluids extricated from animal Subftances by Diftillation and Putrefaction; together with fome Remarks on fulphureous hepatic Air. By* Adair Crawford, *M. D. F. R. S.* — Vide *Philofophical Tranfactions of the Royal Society of London*, Vol. LXXX. *for the Year* 1790, Part II. 4to. London, 1790.

IN the prefent improved ftate of chemical knowledge, great advantages may be expected pected to accrue from a judicious application of it to pathological purpofes. It is with pleafure, therefore, that we fee the ingenious and refpectable author of the paper before us attempting to throw light on the nature of cancer by inquiring into the properties of its matter.

He begins with pointing out the varieties he has remarked in the colour and confiftence of this matter as difcharged by cancerous ulcers. In fome cafes, he obferves, it is of a pale afh colour; in others, of a reddifh caft; and in many inftances of more or lefs of a brown tinge, which fometimes approaches nearly to black. Its confiftence he has found to be, for the moft part, thin; but in the cancerous, as well as in other malignant ulcers, he has frequently met with a white fordes, which clofely adhered to the furface of the fore, and feemed to be fcarcely mifcible with water. In the fame patient the appearance of the difcharge is frequently varied by internal remedies, or by external applications; but if we except the temporary variations produced by accidental circumftances, the cancerous ulcer, in its advanced ftage, is, he obferves, very generally accompanied with a peculiar odour, more highly fetid and offenfive than that which is emitted by other malignant ulcers. It is, he farther remarks, well known that the cancerous matter occafions, by its abforption, fchirrous tumours of the lymphatic glands contiguous to the parts affected; and that it gradually corrodes the branches of the larger blood veffels, which have a peculiar power of refifting the action of other purulent difcharges.

The matter employed by Dr. Crawford in his firft experiments was procured from a patient who had for feveral years been afflicted with a cancer in the breaft. It had been imbibed by cotton, and was of a brownifh caft. Having diffufed it through pure water, he divided it into three parts. To one of thefe he added vegetable fixed alkali; to the fecond, vitriolic acid; and to the third, fyrup of violets. By the vegetable fixed alkali no fenfible change was produced; upon the addition of the vitriolic acid the liquor acquired a deep brown colour, a brifk effervefcence took place, and at the fame time the peculiar odour of the cancerous matter was greatly increafed, and diffufed itfelf to a confiderable diftance through the furrounding air: the fyrup of violets communicated to the portion of liquor to which it was added a faint green colour.

Mr.

Mr. Geber has ſhewn, that animal ſubſtances upon their firſt putrefaction do not effervefce with acids; that, after the proceſs has continued for ſome time, a manifeſt effervefcence takes place; and that this effect again diſappears before the putrefaction has ceaſed.

Suſpecting that the effervefcence in the preceding experiment might have ariſen from a change which the matter underwent, in conſequence of its having been kept ſome days before the trial was made, Dr. Crawford repeated the experiment with a portion of reddiſh matter recently obtained from a cancerous penis. Upon the addition of the acid, the liquor, we are told, acquired, as before, a brown colour, its fetor was much increaſed, and a manifeſt effervefcence took place, although it was not ſo conſiderable as in the former inſtance. A portion of the ſame matter diffuſed through diſtilled water communicated a blue tinge to tincture of litmus, and a greeniſh caſt to ſyrup of violets.

The author obſerves, that when ſyrup of violets was mixed with portions of cancerous matter from a variety of different ſubjects, the change produced was in ſome caſes ſcarcely perceptible; but he aſſures us, that in every in-

ſtance

ſtance the preſence of an alkali was detected by dipping into the matter a ſlip of paper that had been previouſly tinged blue by tincture of litmus, and afterwards ſlightly reddened by acetous acid, the red colour having been invariably in the courſe of a few minutes aboliſhed, and the blue reſtored.

The cancerous matter, as we have already ſeen, acquired, upon the addition of the vitriolic acid, a brown hue. It is well known, that this acid, when it is highly concentrated, communicates a brown or black colour to all animal and vegetable ſubſtances. Being deſirous of learning whether the change which took place upon the addition of the acid to the cancerous matter in this experiment, was different from that which would be produced by the ſame acid in other animal ſubſtances, and particularly in recent healthy pus; Dr. Crawford took equal quantities of the latter, and of aſh-coloured cancerous matter, and having diffuſed each of them through thrice its weight of diſtilled water, he added to them equal quantities of concentrated vitriolic acid; the weight of the acid being nearly the ſame with that of the matter uſed in the experiment. The mixture containing the pus acquired from the acid a faint brown colour; colour; but that which contained the cancerous matter, was fuddenly changed to a deep brown, approaching to black. When thefe mixtures were diluted with about twice their weight of diftilled water, the brown tinge of the former entirely difappeared; but the latter ftill retained its brown colour, although it was fomewhat fainter than it had been upon the firft addition of the acid.

The aërial fluid which was difengaged in the foregoing trials from the matter of cancer, by the vitriolic acid, appeared, we are told, from its odour, to have a nearer refemblance to hepatic than to any other fpecies of air. As it feemed, from its fenfible qualities, to be a very active, and probably a deleterious principle, our author endeavoured more particularly to enquire into its nature, and to compare it with common hepatic air.

The experiments he defcribes as having been made for this purpofe prove, in general, that the fetid odour of the matter of cancer is increafed by the vitriolic, but entirely deftroyed by the concentrated nitrous and dephlogifticated marine acids; that the aërial fluid, which is difengaged by the vitriolic acid, is foluble in water; and that the folution depofits a reddifh brown ptecipitate upon the addition of nitrated ſilver. Whence it follows, that the cancerous matter contains a principle which has many of the properties of hepatic air, and which our author thinks, may perhaps not improperly be termed animal hepatic air.

Having learned from his experiments, as we have ſeen, that the matter of cancer is impregnated with an alkali, which is in ſuch a ſtate as to change the colour of vegetable tinctures, he had very little doubt that this was the volatile alkali; it being well known that putrid animal ſubſtances frequently abound with that ſalt, but have never been found to contain a fixed alkali in a diſengaged ſtate. With a view, however, more deciſively to determine this point, he tried the following experiment:—A quantity of cancerous matter, diffuſed through diſtilled water, was introduced into a glaſs retort to which a receiver was adapted. The mixture was ſlowly diſtilled by means of a ſand heat; and a ſmall quantity of the liquor which came over into the receiver being poured into an infuſion of Brazil wood, inſtantly imparted to it a deep red colour.

Hence it clearly appeared, that the alkali contained in the cancerous matter was the volatile, tile, becaufe it was feparated by diftillation with a heat which did not exceed that of boiling water.

It feemed extremely probable, that the above-mentioned alkali was united to the aërial fluid with which the matter of cancer is impregnated. Of the truth of this fact Dr. Crawford was perfuaded by obferving, that the fmell of the cancerous matter was greatly increafed by the addition of the vitriolic acid: for he could fcarcely avoid concluding, that this phænomenon arofe from an union between the acid and alkali, in confequence of which the odoriferous principle was extricated by a fuperior attraction.

This conclufion, he obferves, has been confirmed by his fubfequent experiments, which prove that the volatile alkali is capable of combining chemically with the aërial fluid contained in the matter of cancer.

Dr. Crawford next treats of the air extricated from cancerous matter, and from other animal fubftances, by diftillation.

A portion of matter from a cancerous breaft was diffufed through diftilled water, and introduced into a fmall coated glafs retort, which was gradually expofed to heat in a fand bath till the bottom of the retort became red-hot. The neck neck of the latter was introduced below an inverted jar filled with water, and a quantity of air was received in the jar, which was found to confiſt of the common air contained in the retort. Two meaſures of it, mixed with one of nitrous air, occupied the ſpace of a little leſs than two meaſures. This portion of air was ſtrongly impregnated with the peculiar ſmell of the cancerous matter.

The heat continuing to increaſe, the water began to boil, and a large quantity of aqueous vapour aroſe; which, as ſoon as it came into contact with the common air, produced a white ſmoke. The ſmell that was now perceived was remarked by thoſe who were preſent to be ſimilar to that of freſh animal ſubſtances when they are boiled. The aqueous vapour in this part of the proceſs was not mixed with any permanently elaſtic fluid.

When the greater part of the water was evaporated, the jar containing the firſt portion of air was removed, and the neck of the retort was introduced beneath an inverted veſſel filled with mercury. Soon after this, a conſiderable quantity of air, having a fetid ſmell ſimilar to that of burned bones, was extricated. This aërial fluid was mixed with a yellow empyreumatic matic oil. A portion of it being agitated with water was found to be partly imbibed by that fluid; and nitrated ſilver, dropped into the water thus impregnated, produced a reddiſh precipitate.

One meaſure of the air, obtained in the foregoing experiment, being mixed over mercury with an equal bulk of alkaline air, the volume of the mixture was found gradually to decreaſe; and, at the end of three hours, the air in the tube occupied the ſpace of only one meaſure and two tenths. An oily depoſit was now made upon the inner ſurface of the tube. At the expiration of eight days, the interior ſurface of the tube was covered with ſlender films, which had a yellowiſh caſt, and which were irregularly ſpread upon it. The upper ſurface of the mercury within the tube was corroded; in ſome places it had a reddiſh burniſhed appearance; in others, it was changed into an aſh-coloured powder, interſperſed with brown ſpots. The tube was now removed from the mercury, and the air that remained in it had a ſtrong fetid ſmell, reſembling that of burned bones.

From theſe experiments, the author obſerves, it appears that the matter upon which the peculiar liar ſmell of cancerous ulcers depends is a very volatile ſubſtance, for it eſcaped at the beginning of the proceſs; and alſo that this volatile ſubſtance, which, he thinks, is probably the active principle in the matter of cancer, is not changed, by ſimple expoſure to heat, into a permanently elaſtic fluid; for the air that eſcaped at the beginning of the proceſs, although it ſmelled ſtrongly of the cancerous matter, was found, it ſeems, by Dr. Prieſtley's teſt, to be as pure as common air: and it was evident, he remarks, that the aqueous vapour which came over in the middle of the proceſs was not mixed with any permanently elaſtic fluid; becauſe when this vapour was received in an inverted bottle filled with mercury, it was condenſed into water without any admixture of air.

From ſubſequent experiments with the fleſh of a chicken, and with putrid veal, which the author deſcribes, we learn that the aërial fluids extricated from freſh as well as from putrid animal ſubſtances, by diſtillation, have nearly the ſame properties with that which is diſengaged, by a ſimilar proceſs, from the matter of cancer. Each of them appears to conſiſt of two diſtinct fluids; one of which is ſoluble, and and the other insoluble, in water. Dr. Crawford observes of the insoluble portion, that it burns with a lambent flame, and has all the characters of heavy inflammable air; whereas the soluble part, he tells us, resembles the fluid which is extricated from cancerous matter by the vitriolic acid: it has a fetid odour, it decomposes nitrated silver, combines with caustic volatile alkali, and possesses many of the properties of common hepatic air.

There are, however, he observes, several particulars in which the animal and common hepatic air materially differ from each other. Although they are both fetid, yet their odours are not exactly similar. When common hepatic air is decomposed by the concentrated nitrous or dephlogisticated marine acid, sulphur is separated; but when animal hepatic air is decomposed by these acids, a white flaky matter is disengaged which is evidently an animal substance, because it becomes black by the addition of concentrated vitriolic acid. Sulphur is moreover separated during the combustion of common hepatic with atmospherical air; but when the air from animal substances is burned with atmospherical air, no precipitation of sulphur takes place. Indeed, that animal hepatic air does not contain ſulphur will be apparent, he thinks, from the following experiment.

Equal parts of pure air and of air extricated from freſh beef by diſtillation, were fired by the electric ſhock in a ſtrong glaſs tube over mercury. A little diſtilled water was then introduced through the mercury into the tube, and was agitated with the air which it contained. A portion of this water being filtered, and a ſmall quantity of muriated barytes being dropped into it, the mixture remained perfectly tranſparent. Hence, he infers, that the air extricated from freſh beef by diſtillation does not contain ſulphur; for, if it had contained that ſubſtance, the ſulphur, by its combuſtion with the pure air, would have been changed into the vitriolic acid, and the muriated barytes would have been decompoſed.

He obſerves, that he has frequently repeated the preceding experiment with the air extricated, by diſtillation, from the putrid as well as from the freſh muſcular fibres of animals, but without having been able to diſcover the leaſt veſtige of vitriolic acid.

The experiments, of which the author next gives an account, were made with a view to analyſe the airs which are diſengaged from animal animal ſubſtances by heat. From theſe it appears that the ſoluble part of the air which is diſengaged from the lean of animal ſubſtances, by heat, conſiſts of three diſtinct fluids, viz. of alkaline air, fixed air, and animal hepatic air.

After deſcribing ſome experiments made with a view to determine the products which reſult from the combuſtion of pure air with animal air, or with the compound aërial fluid extricated from the lean of animal ſubſtances by heat, Dr. Crawford proceeds to treat of the products which reſult from the combuſtion of ſulphureous hepatic with pure air; of the air extricated from animal ſubſtances by putrefaction, (and which, from his experiments, appears to conſiſt of fixed and animal hepatic, mixed with a very ſmall proportion of phlogiſticated air); and laſtly, of the effects produced by expoſing freſh animal ſubſtances to atmoſpherical, hepatic, and pure air. The experiments related under the latter of theſe heads appear, he thinks, to lead to the following concluſions reſpecting the proceſs of putrefaction in the lean of animal ſubſtances:

"The muſcular fibres of animals contain "fixed and phlogiſticated air, the inflamma-

" ble principle in the ſtate of heavy and of

" light inflammable air, and a ſubſtance which,

" by means of heat or of putrefaction, is capa-

" ble of being converted into animal hepatic

" air *. When the muſcular fibre, after the

" death of the animal, is expoſed to the pure

" air of the atmoſphere, the latter, by a ſu-

" perior attraction, combining with the heavy

" inflammable air, produces fixed air, and at

" the ſame time furniſhes the quantity of heat

" neceſſary to the formation of animal hepatic

" air. The coheſion of the fibre being thus

" deſtroyed, the fixed, as well as the light in-

" flammable and phlogiſticated air, which enter

" into its compoſition, are diſengaged, and the

" two latter fluids uniting with each other pro-

" duce the volatile alkali.

" The alterations which take place in putre-

" faction are in moſt reſpects ſimilar to thoſe

" which ariſe from deſtructive diſtillation. By

" expoſure to heat the fixed air of the animal

* In a note to this part of his paper the author obſerves, that the exiſtence of fixed, inflammable, and phlogiſticated air in animal ſubſtances, and the compoſition of volatile alkali, were diſcovered before he began to give particular attention to this ſubject.

" fibre

" fibre is extricated, hepatic air and volatile al-
" kali are produced, and the inflammable prin-
" ciple not coming into contact with the pure
" air of the atmosphere, is raised in the form
" of heavy imflammable air."

Dr. Crawford observes, that, from the experiments he has related, it appears that in cancerous and other malignant ulcers, the animal fibres undergo nearly the same changes which are produced in them by putrefaction, or by destructive distillation. The purulent matter prepared for the purpose of healing the ulcer is, in such cases, mixed with animal hepatic air and volatile alkali. The compound formed by the union of these substances, which he thinks, may perhaps not improperly be termed hepatised ammonia, decomposes metallic salts, and acts upon metals.

These facts, he observes, seem to afford an explanation of the changes produced in metallic salts, when they are applied to malignant ulcers; the volatile alkali combining with the acid of the metallic salt, and the animal hepatic air reviving the metal, either by imparting to it the inflammable principle, or by uniting with the pure air which the calx is supposed to contain. He thinks it probable that the metal, thus revived, is in ſome caſes again corroded by the hepatiſed ammonia, which communicates to it a black colour. Thus we may account, he obſerves, for the dark incruſtation frequently formed upon the tongue and internal fauces, when venereal ulcers of the throat are waſhed with a ſolution of corroſive ſublimate. And hence alſo, according to our author, the dark tinge which is frequently communicated by ill-conditioned ulcers to poultices made with a ſolution of ſugar of lead. The action of the hepatiſed ammonia, he farther remarks, explains the reaſon why the probes are frequently corroded when they are introduced into ſinuous ulcers, or applied to the ſurfaces of carious bones; and to the ſame cauſe he thinks it is probably owing, that poliſhed metallic veſſels are quickly tarniſhed, when they are expoſed to the effluvia of putrid animal ſubſtances.

From his experiments, the author obſerves, it appears, that animal hepatic air imparts to the fat of animals recently killed a green colour; that it renders the muſcular fibres ſoft and flaccid, and increaſes the tendency to putrefaction. It is therefore a ſeptic principle, and hence he conſiders it as extremely probable, that the compound of this fluid with volatile alkali, which which is found in the matter difcharged by the open cancer, produces deleterious effects; for although the mifchief in cancerous ulcers feems principally to depend upon a morbid action of the vefels, whence the unhealthy ftate of the matter difcharged by fuch ulcers is fuppofed to derive its origin, yet from the corrofion of the coats of the larger blood-veffels, and the obftructions in the contiguous glands, there can, he thinks, be little doubt that this matter aggravates the difeafe. The experiments he has recited, appear to prove, that the hepatifed ammonia is the ingredient which communicates to the cancerous matter its putrid fmell, its greater thinnefs, and, in a word, all the peculiar properties by which it differs from healthy pus.

From thefe confiderations he inferred, that a medicine which would decompofe the hepatifed ammonia, and deftroy the fetor of the animal hepatic air, without at the fame time increafing the morbid action of the veffels, would be productive of falutary effects. The nitrous acid, he obferves, does not deftroy the fetor of hepatic air, unlefs it be highly concentrated; and in this ftate it is well known that it fpeedily corrodes animal fubftances. But the fetor of hepatic air quickly diſappears when it is mixed with the dephlogiſticated marine acid, even though the latter be ſo much diluted with water as to render it a very mild application. He has found that this acid, diluted with thrice its weight of water, gives but little pain when it is applied to ulcers that are not very irritable; and in ſeveral caſes of cancer it appeared to correct the fetor, and to produce a thicker and more healthy pus. He very candidly adds, however, that other caſes occurred in which it did not ſeem to be attended with the ſame ſalutary effects. He remarks at the ſame time that ſome cancerous ulcers are ſo extremely irritable, that applications which are at all of a ſtimulating nature cannot be ventured upon with ſafety. And hence if the obſervations, which he has made on the efficacy of this acid as an external application, ſhould be confirmed by future experience, he leaves it to the judgment of the ſurgeon to determine both the degree of its dilution, and the caſes in which it may be employed with advantage.

The dephlogiſticated marine acid, as is generally known, has the power of deſtroying the colour, the ſmell, and perhaps the taſte, of the greater part of animal and vegetable ſubſtances.

We have feen that it corrects the fetor of putrid flefh. And our author has found, that, when it is poured in fufficient quantity upon hemlock and opium, thefe narcotics fpeedily lofe their fenfible qualities. As it appears, therefore, to poffefs the power of correcting the vegetable, and probably many of the animal poifons, it feemed not unlikely, that it might be ufeful as an internal medicine. Conceiving that its exhibition would be perfectly fafe, he once took twenty drops of it diluted with water. He foon afterwards, however, felt an obtufe pain, with a fenfe of conftriction in his ftomach and bowels. This uneafinefs, notwithftanding the ufe of emetics and laxatives, lafted for feveral days, and was at length removed by drinking water impregnated with fulphureous hepatic air. He afterwards found, that the manganefe, which had been ufed in the diftillation of the acid, contained a fmall portion of lead.

Dr. Ingen-houfz has informed him, that a Dutchman of his acquaintance, fome time ago, drank a confiderable quantity of the dephlogifticated marine acid : the effects which it produced were fo extremely violent, that he nearly efcaped with his life. If therefore this acid fhould hereafter be employed as an internal medicine, it would be be neceſſary, our author thinks, to prepare it by means of manganeſe that has been previouſly ſeparated, by a chemical proceſs, from the lead and other metals with which that ſubſtance is uſually contaminated.

